# Heterozygous loss‐of‐function alleles associate the conserved 3′‐5′ exoribonuclease EXOSC10 with hypersensitivity to the anticancer drug 5‐fluorouracil

**DOI:** 10.1002/1878-0261.70239

**Published:** 2026-05-15

**Authors:** Radhika Sain, Yann Le Page, Frédéric Percevault, Emmanuelle Becker, Luc Negroni, Almudena Fernandez, Marta Cantero, Diego Muñoz‐Santos, Lluís Montoliu, Cyrille Garnier, Michael Primig

**Affiliations:** ^1^ Inserm, EHESP, Irset (Institut de recherche en santé, environnement et travail) ‐ UMR_S 1085 Univ Rennes Rennes France; ^2^ ESBS, ‐BCS‐UMR7242 Illkirch France; ^3^ National Centre of Biotechnology (CNB‐CSIC) Madrid Spain; ^4^ Biomedical Research Networking Center on Rare Diseases (CIBERER‐ISCIII) Madrid Spain; ^5^ Department of Medicine. Faculty of Medicine, Health and Sports Universidad Europea de Madrid Madrid Spain; ^6^ Present address: Univ Rennes, Inria, CNRS, IRISA Rennes F‐35000 France

**Keywords:** 5‐fluorouracil, EXOSC10, Rrp6, ubiquitination

## Abstract

The 3′‐5′ exoribonuclease EXOSC10 degrades aberrant mRNAs and noncoding RNAs in cooperation with the nuclear RNA exosome. EXOSC10's localization and stability are regulated by sumoylation and proteasomal degradation in response to stress, and the protein is essential for cell growth and proliferation, fertility, hematopoiesis, and brain development. EXOSC10 is a cancer biomarker; its activity is inhibited by the widely used anticancer drug 5‐fluorouracil (5‐FU) and the protein's depletion sensitizes cells to 5‐FU. We employed mass spectrometry to reveal EXOSC10's post‐translational modifications, such as phosphorylation, acetylation and ubiquitination, and to explore its protein interaction network, which includes RNA exosome subunits and enzymes involved in protein degradation. Furthermore, we find that the *EXOSC10* ^S402T^ allele identified in colon cancer and located within a motif for targeted proteolysis is stable, nuclear but nonfunctional *in vivo*, since homozygous *Exosc10* ^S402T^ mice exhibit early embryonic lethality. We identified equivalent S402P/S402A variants and heterozygous loss‐of‐function (LoF) alleles in cancers and healthy individuals using public genomics data. Our findings suggest that recessive *EXOSC10* LoF alleles may cause increased 5‐FU sensitivity in tumors bearing *de novo* mutations and hypertoxicity in heterozygous carriers.

## Graphical abstract

EXOSC10, an essential nuclear RNA exosome‐associated 3′‐5′ exoribonuclease, is inhibited by the anticancer drug 5‐fluorouracil (5‐FU), and EXOSC10 depletion increases 5‐FU sensitivity. The colon‐cancer variant EXOSC10^S402T^, located in a proteolysis motif, is stable and nuclear but nonfunctional *in vivo*. Recessive EXOSC10 loss‐of‐function alleles are potentially associated with elevated tumor 5‐FU sensitivity and 5‐FU hypertoxicity in heterozygous carriers.

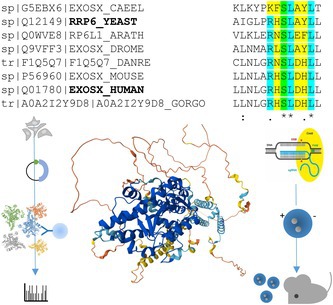

**Abbreviations**
5‐FU: 5‐fluorouracil
APC/C: Anaphase promoting complex/cyclosome
COSMIC: Catalog of Somatic Mutations in Cancer
DDA: Data‐Dependent Acquisition
ELM: Eucaryotic Linear Motif
EXO1: DEDDy 3′‐5′ exonuclease domain
FDR: False discovery rate
Fs: Frameshift allele
gnomAD: The genome Aggregation Database
HRDC: Helicase and RNase D C‐terminal domain
lncRNAs: long noncoding RNAs
LoF: Loss‐of‐function
MS: Mass Spectroscopy
MSA: Multiple sequence alignment
PM/Scl: Polymyositis/scleroderma overlap syndrome
PRIDE: Proteomics Identifications Database
PSM: Peptide spectrum match
PTM: Post‐translational modification
SNV: Single nucleotide variant
Ter: Termination allele
WCE: Whole cell extract

## 1 Introduction

The conserved 3′‐5′ exoribonuclease EXOSC10/Rrp6 is mainly involved in degrading and processing aberrant mRNAs and noncoding RNAs as one of the catalytic subunits of the nuclear RNA exosome [1, 2]. In addition, it has been reported to function in cooperation with various co‐factors [3, 4], and also alone via its own RNA binding domain [5]. The protein consists of an N‐terminal region that contains the PMC2NT domain needed for interacting with C1D|Rrp47 [6, 7], an internal region comprising the catalytic *DEDDy 3′‐5′ exonuclease* (EXO1) and *helicase and RNase D C‐terminal* (HRDC) domains [8], and a C‐terminal region that binds RNA, activates the nuclear RNA exosome and contains a nuclear localization signal [5, 9]. Earlier molecular biological and functional genomics analyses identified EXOSC10 as a major hub protein that physically interacts with 241 proteins (as of March 2026; http://thebiogrid.org) [10].

Several lines of evidence point to mechanisms controlling the stability of EXOSC10/Rrp6: human EXOSC10 is negatively regulated by low‐temperature stress in a sumoylation‐dependent manner, and EhRrp6 from the protozoan parasite *Entamoeba histolytica* is depleted by growth stress via a proteasome‐dependent mechanism [11, 12]. High‐throughput studies have identified a large number of ubiquitinated proteins, including yeast Rrp6 and its mammalian ortholog EXOSC10 [13]. However, the functional significance of EXOSC10's ubiquitination in the context of cell division, development, and disease remains to be understood, given that the protein declines during meiotic differentiation [14, 15], but does not appear to be regulated during the mitotic cell cycle [16]; reviewed in [17].

The anaphase promoting complex/cyclosome (APC/C) is a conserved multimeric ubiquitin ligase that targets proteins for destruction by the proteasome, and thereby couples progression through mitotic cell division with developmental processes; reviewed in [18]. Importantly, the complex is also involved in the heat shock response, cell differentiation, and metabolism [19, 20]. The APC/C recognizes its substrates via so‐called degrons that are organized into destruction boxes (D‐boxes) and KEN boxes, depending on their highly conserved amino acid sequences; reviewed in [21].

Previous work in yeast, fly, and mouse identified EXOSC10/Rrp6 target RNAs [15, 22, 23, 24, 25, 26, 27, 28], reported functions in rRNA processing—notably of 5.8S RNA, which is important for ribosome translocation [9, 22, 29], and revealed a role in controlling long noncoding RNAs (lncRNAs) involved in DNA double strand break repair [30, 31]. Global and tissue‐specific *in vivo* analyses revealed essential roles for mouse *Exosc10* during early embryogenesis, male and female gametogenesis, erythropoiesis, B‐cell gene expression, and brain development [14, 26, 32, 33, 34, 35, 36]. Five loss‐of‐function (LoF) alleles lacking catalytic activity were identified in yeast *RRP6* [37], and four of these LoF mutations were reported to be conserved in the human ortholog *EXOSC10* [38].

Human EXOSC10 is of clinical interest because it has been associated with an autoimmune condition called the polymyositis/scleroderma overlap syndrome (PM/Scl, hence its initial name PM/Scl‐100) [39, 40, 41]; for review, see [17]. The protein is also important for molecular oncology because it is essential for mitotic division of cultured cancer cells [42], it is a potential prognostic marker for liver and thyroid cancer [43], and it functions as a tumor suppressor in bladder urothelial carcinoma [44]. Intriguingly, the widely used anticancer drug 5‐fluorourcil (5‐FU) inhibits EXOSC10's exoribonucleolytic activity, and was recently found to exert its cytotoxic activity predominantly via an RNA‐dependent pathway important for ribosome biogenesis [45, 46]. It is remarkable that cells depleted of EXOSC10 are hypersensitive to 5‐FU because it suggests a direct implication of the protein's cellular level in the cytotoxic mechanism of the drug [47, 48]. Finally, the Catalog of Somatic Mutations in Cancer (COSMIC; https://cancer.sanger.ac.uk/cosmic) and the genome Aggregation Database (gnomAD; https://gnomad.broadinstitute.org) list a large number of likely deleterious missense, termination, and frameshift mutations located upstream of or within critical functional domains of EXOSC10 [49, 50].

We hypothesized that EXOSC10/Rrp6 alleles with altered stability or activity may influence 5‐FU cytotoxicity. To test this, we scanned human EXOSC10 for the extended D‐box motif RxxLxx[LIVM] and identified a highly conserved sequence. A search for mutations within this motif in malignant tumors revealed a serine‐to‐threonine substitution at Position 402 (S402T) in a somatic cancer sample (cosmic) [51]. Mass spectrometry of affinity‐purified EXOSC10 identified phosphorylated residues, ubiquitinated lysines, and APC/C subunits among co‐immunoprecipitated (Co‐IP) proteins. Molecular analyses of EXOSC10^S402T^ in cancer cells revealed that the mutant allele is stable and not targeted by the APC/C‐proteasome pathway under the conditions tested. Using publicly available data, we identified equivalent S402P and S402A missense mutations, along with numerous predicted frameshift and termination LoF alleles in heterozygous carriers and various cancers. Critically, homozygous *Exosc10* ^S402T^ mice displayed early embryonic lethality, similar to effects observed in our previous gene deletion study [34]. We propose that *EXOSC10* ^S402T^ is a recessive LoF allele, potentially associated with increased 5‐FU sensitivity in tumors harboring similar LoF mutations, as low EXOSC10 levels enhance 5‐FU toxicity. These findings have possible therapeutic implications for treating tumors with heterozygous *EXOSC10* LoF alleles, and for cancer patients carrying these rare but potentially fatal mutations when undergoing 5‐FU‐based chemotherapy.

## 2 Materials and methods

### 2.1 Bioinformatics tools

The motif search was carried out using scanprosite (https://prosite.expasy.org) [52]. Multiple protein sequence alignments were performed with the Multiple Sequence Comparison by Log‐Expectation tool provided by the European Bioinformatics Institute (muscle, https://www.ebi.ac.uk/jdispatcher/msa/muscle) [53]. The effect of missense mutations on protein structure and function was predicted using the Polymorphism Phenotyping algorithm (PolyPhen‐2, http://genetics.bwh.harvard.edu/pph2/) and alphamissense (https://console.cloud.google.com/storage/browser/dm_alphamissense) [54, 55]. The impact of mutations on protein dynamics and stability caused by changes in vibrational entropy was analyzed and visualized using the dynamut server (https://biosig.lab.uq.edu.au/dynamut/) [56].

### 2.2 Data sources and knowledgebases

The D‐box consensus was provided by the Eukaryotic Linear Motif resource (elm; http://elm.eu.org) [57]. Genetic data for normal tissues were retrieved from the genome Aggregation Database (gnomad; https://gnomad.broadinstitute.org), and for cancer samples from cosmic (http://cancer.sanger.ac.uk/cosmic) [49, 50] and cbioportal (https://www.cbioportal.org) [58]. Information was interpreted about resolved protein structures provided by the Protein Databank (PDB; https://www.rcsb.org) [59], protein structure predictions from the EBI's alphafold database (https://alphafold.ebi.ac.uk) [60], conserved protein domains, motifs, and amino acids from interpro (https://www.ebi.ac.uk/interpro/) [61], post‐translational protein modifications from phosphositeplus (www.phosphosite.org) [62], and protein–protein interaction data from the String database (https://string‐db.org) [63] and The biogrid (www.thebiogrid.org) [10].

### 2.3 Cell lines, cell culture conditions, and cell transfection

HEK293T (RRID: CVCL_0045; ATCC origin, Manassas, VA, USA) and MCF7 (RRID: CVCL_0031; ATCC origin) cells were cultured using standard culture conditions as described previously [64, 65]. Briefly, cells were incubated in Dulbecco's Modified Eagle Medium (DMEM) (41 966–029; Gibco, Thermo Fisher Scientific, Waltham, MA, USA) supplemented with 8% fetal bovine serum (FBS) (S181H‐500; Biowest, Nuaillé, France) and antibiotics (Gibco) at 37 °C in a humidified incubator with 5% CO_2_.

For protein overexpression, cells were transfected with an empty control vector, a vector harboring wild‐type CMV‐EXOSC10^DKK‐MYC^, and plasmids harboring missense mutants (S402T, K583R) using jetpei (101 000 020; Polyplus, Illkirch, France) according to the manufacturer's instructions. For *C1D* siRNA depletion experiments, cells were transfected with a mixture of three unique 27mer duplex siRNAs (SR307080; OriGene, Herford, Germany) or with nonspecific control siRNA. The optimal siRNA concentrations were determined according to the manufacturer's instructions. Here, cells were transfected with jetprime® (101 000 027; Polyplus) according to the manufacturer's instructions at a final siRNA concentration of 10 nm. During transfection, we maintained the cells in phenol red‐free DMEM (31 053–028; Gibco) containing 2.5% charcoal‐stripped FBS (S181W‐500; Biowest) for at least 24 h, before the medium was replaced with DMEM supplemented with 8% FBS (S181H‐500; Biowest) and antibiotics, during continued incubation for 48–72 h. Cell lines are regularly authenticated via morphological features and physiological criteria, such as hormone responsiveness. All experiments were performed with mycoplasma‐free cells.

### 2.4 Protein affinity purification

HEK293T (RRID:CVCL_0045; ATCC origin) cells were cultured under standard conditions and transfected them with JetPEI® (101 000 020; Polyplus) using a vector harboring CMV‐*EXOSC10* ^MYC‐DDK^ (RC210055; OriGene). After 72 h, 2 × 10^8^ cells were harvested, washed with 1 × PBS, and lysed in ice‐cold RIPA buffer (10 mm Tris–HCl pH 8, 1 mm EDTA, 0.5 mm EGTA, 1% Triton X‐100, 140 mm NaCl, 1 × Protease inhibitor). The lysate was pipetted 20 times to ensure homogenous lysate and centrifuged at 14000 ***g*** for 15 min at 4 °C to separate the extract from the cell debris. Anti‐FLAG M2 Magnetic beads (M8823; Millipore, Molsheim, France) were equilibrated in 1 × TBS buffer (50 mm Tris–HCl, 150 mm pH 8) before incubating them over night at 4 °C with 2.7 μg total protein extract. The beads were washed three times with 1 × TBS buffer and four times with 1 × PBS. Finally, beads were stored at −20 °C until the sample was processed for MS‐analysis.

### 2.5 Protein identification by mass spectrometry

Proteins retained on the beads were digested for 4 h with 250 ng trypsin (Promega, Madison, WI, USA) in 0.1 m Tris pH8, 2 mm CaCl_2_. The supernatant (40 μL) was transferred into the LC injection vial and 4 μL 200 mm DTT was added before loading into the LC autosampler. LC–MS/MS was carried out with Ultimate 3000 nano‐RSLC coupled in line with an orbitrap Exploris 480 via a nano‐electrospray ionization source and the FAIMS pro interface (Thermo Fisher Scientific). A maximum of 200 ng of sample was loaded on a C18 Acclaim PepMap100 trap‐column (75 μm ID × 2 cm, 3 μm, 100 Å; Thermo Fisher Scientific) for 3.5 min at 5 μL·min^−1^ with 2% ACN, 0.1% FA in H2O and then separated on a C18 Accucore nano‐column (75 μm ID × 25 cm, 2.6 μm, 150 Å; Thermo Fisher Scientific) with a 60 min linear gradient from 8% to 20% buffer B, a 16 min gradient from 20 to 30 followed by a regeneration step at 90% B and a re‐equilibration at 8% B (A: 0.1% FA in H2O; B: 0.1% FA in 80% ACN, 450 nL·min^−1^, 50 °C). The total chromatography time was 90 min. The mass spectrometer was operated in positive ionization mode in Data‐Dependent Acquisition (DDA) with FAIMS compensation voltage set to CV = –55 V. The DDA cycle consisted of one survey scan (330–1400 m/z, 90 000 FWHM) followed by MS^2^ within 1.3 s (HCD, 30% normalized energy, 8 m/z window, 22 500 FWMH). Unassigned and single charged states were rejected. Ion exclusion was set for 30 s with a 10 ppm mass width. MS raw files were processed with the Proteome Discoverer 2.5 software (Thermo Fisher Scientific), and *Homo sapiens* swissprot proteome database (release 03 August 2024, 20 597 sequences) implemented with a contaminants database and the primary sequence of human EXOSC10. Precursor and fragment mass tolerances were set at 7 ppm and 0.02 Da, respectively, and up to two missed cleavages were allowed. Oxidation (*M*), pyro‐glu N (N‐terminal *Q*), Met‐loss, Met‐loss+Acetyl, Acetyl (N terminus) was set as variable modifications. Peptides and proteins were filtered with a false discovery rate (FDR) at 1%. Proteins were quantified with a minimum of 1 unique peptide based on extracted ion chromatogram (XIC).

### 2.6 EXOSC10 post‐translational modification (PTM) analysis by mass spectrometry

To identify EXOSC10 PTMs additional optional modifications phospho (S, T, Y), GG ubiquitin remnant motif (K), acetyl (K), and methyl (K) were added to the analysis procedure in Proteome Discoverer 2.5. The PTM position site probability was assessed with IMP‐ptmRS in Proteome Discoverer.

### 2.7 Site‐directed mutagenesis

To generate *EXOSC10* S402T and K583R missense mutations, the pCMV6 *EXOSC10* ^MYC‐DDK^ vector (RC210055; OriGene) was employed as a template for the QuikChange II site‐directed mutagenesis kit (200 521; Agilent, Santa Clara, CA, USA). Oligonucleotide forward and reverse primers were designed containing the c.1204T>A substitution mutation for S402T (t1204a_f: 5′ ACC‐TGG‐GCA‐GGC‐ACA‐CAC‐TCG‐ATC‐ATC‐TC 3′; t1204a_r: 5′ GAG‐ATG‐ATC‐GAG‐TGT‐GTG‐CCT‐GCC‐CAG‐GT 3′) and the c.1748A>G substitution mutation for K583R (a1748g_f: 5′ AGA‐TGC‐CCC‐TGC‐TCA‐GGT‐CTG‐AAG‐TTG‐CAG‐C 3′; a1748g_r: 5′ GCT‐GCA‐ACT‐TCA‐GAC‐CTG‐AGC‐AGG‐GGC‐ATC‐T 3). The primers were purified by the service provider using the RP‐cartridge protocol (Eurogentec, Seraing, Belgium). The mutagenesis procedure was performed according to the manufacturer's instructions, and DNA isolated from several resulting clones was sequenced to verify the mutations (Eurogentec).

### 2.8 Protein analysis by Western blotting

2x10^6^ cells were harvested, washed, and lysed in 1 × Laemmli buffer (6% SDS) before total protein samples were separated using 8.5% SDS polyacrylamide gels prior to transferring them onto a nitrocellulose membrane (Protran, cat. no.: 10600002; Cytiva Amersham, UK) using a semi‐dry Trans‐Blot electroblotter (Bio‐Rad, Hercules, CA, USA). The membranes were blocked in 5% dry milk before adding the primary anti‐EXOSC10 antibody (ab264343; Abcam, Cambridge, UK) at 1:2000 or the anti‐C1D antibody (M03 clone 6H2; Abnova, Taipei City, Taiwan) at 1:1000 overnight at 4°C on a rotary shaker. An anti‐β‐Actin (ACTB) antibody (A1978; Sigma‐Aldrich, Saint Louis, MO, USA) was employed as a loading control at a dilution of 1:000. Signals were detected by incubating the membrane with a secondary anti‐rabbit antibody at 1:5000 (NA934V; Cytiva) or a secondary anti‐mouse antibody (NA9310V; Cytiva) at 1:5000. The signals were revealed by employing Immobilon Western Chemiluminescent HRP substrate (WBKLS0100; Millipore) according to the manufacturer's instructions. Finally, band signal intensities were quantified using the Image Lab software version 5.2.1 (Bio‐Rad).

### 2.9 Quantitative EXOSC10 wild‐type and mutant protein detection by immunocytochemistry

HEK293T cells were seeded on cover slips in 24‐well plates and incubated for 24 h before transfecting them with a vector harboring CMV‐EXOSC10^DKK‐MYC^ (RC210055; OriGene) or the mutant variants CMV‐EXOSC10^DKK‐MYC^ S402T and K583R. After 72 h, the cells were washed twice with 1 × phosphate‐buffered saline (PBS), fixed with 4% paraformaldehyde in 1 × PBS for 20 min, and then washed three times with 1 × PBS. The cells were permeabilized with 0.25% Triton X‐100 in 1 × PBS for 20 min. Following permeabilization, the cells were incubated overnight at 4 °C with the monoclonal anti‐MYC tag primary antibody (9B11 Mouse mAb, no.: 2276; Cell Signaling Technology, Danvers, MA, USA) at a dilution of 1 : 1000 in 1 × PBS containing 3% FBS. For immunofluorescence detection, the cells were incubated with a fluorescent dye‐conjugated secondary antibody at 1 : 1000 (ab150109; Abcam) in 1 × PBS containing 3% FBS for 1 h at room temperature. The cover slips were mounted using duolink ll mounting medium with dapi (DUO82040; Sigma‐Aldrich). Images were obtained using an ApoTome Axio Z1 Imager microscope (zeiss) and processed with the axio Vision Software. A custom macro was developed using the fiji software (https://imagej.net/software/fiji; [66]) for automated quantification of nuclear fluorescence in transfected cells. Fluorescence background and mean nuclear area were determined from a set of randomly selected images. Background fluorescence was subtracted from all images prior to analysis. Nuclear masks were generated using Otsu's thresholding algorithm, and objects with areas outside the defined nuclear size range (mean ± 30%) were excluded. Mean nuclear fluorescence intensity was calculated for each construct from at least 10 images, representing approximately 80–100 cells.

### 2.10 qRT‐PCR assay

Total RNA was isolated using the NucleoSpin RNA Plus kit (Macherey‐Nagel, Hoerdt, France). Five hundred nanograms of total RNA was reverse‐transcribed into cDNA using the iScript Reverse Transcription Supermix Kit (Bio‐Rad, Marnes‐la‐Coquette, France). Quantitative real‐time PCR was performed using the iQ SYBR Green Supermix kit (Bio‐Rad), and samples were analyzed using the CFX384 Real‐Time PCR System (Bio‐Rad). Relative changes in gene expression levels were determined using the standard ΔCt method; the signals were normalized using *GAPDH* and *TBP* (63). Oligonucleotide primers are shown in Table 1.

**Table 1:** qRT‐PCR oligonucleotide primer annotation and sequences. Primer names of forward (F) and reverse (R) oligonucleotides and their sequences are shown.

C1DF	5′‐TTGGCAACCCAAGGAGTTAATCC‐3′
C1DR	5′‐TCTGTCCAGCTTGCCAGCCTTT‐3′
EXOSC10F	5′‐CAGGCCATCTCCGTCCGACA‐3′
EXOSC10R	5′‐TGGCTCTGGGTCCTTTGGCT‐3′
GAPDHF	5′‐TGCACCACCAACTGCTTAGC‐3′
GAPDHR	5′‐GGCATGGACTGTGGTCATGAG‐3′
TBPF	5′‐TGCACAGGAGCCAAGAGTGAA‐3′
TBPR	5′‐CACATCACAGCTCCCCACCA‐3′

### 2.11 Generating genome‐edited *Exosc10* mutant alleles using CRISPR‐Cas9 tools

The formal nomenclature of mice referred to in this manuscript as Exosc10<S402T> is Exosc10<em1>/Cnbc. To obtain mice carrying the c.1204T>A (p.S402T) *Exosc10* allele (mutation ID: COSM1332574, GRCh38, COSMIC v84) in exon 10, the coding regions and deduced protein sequence for the human and mouse EXOSC10 genes were aligned. In both cases, the nucleotide and amino acid numbering coincided; therefore, the design of the guide RNA (gRNA) for the CRISPR‐Cas9 approach was initiated using the Breaking‐Cas bioinformatic web program developed at CNB [67]. The selected sgRNA (5′‐GGTCGAGTGAGTGCCGAGCC‐3′) was associated with a compatible PAM sequence (AGG) and verified not to be associated with significant predicted off‐target events on chromosome 4 where *Exosc10* is located. The expected mutation was positioned in the middle of the sgRNA sequence. To promote the indicated mutation, a single‐strand oligodeoxynucleotide (ssODN, 92mer) was synthesized to be used as donor DNA sequence: 5′‐TGAACATGTTTGACACACACCAGGCAGCACGGCTTCTCAACCTGGCTCGGCACACACTCGACCATCTGCTGAGACTCTACTGCGGTGTGGAA‐3′. This ssODN included the c.1204T>A mutation (Position 54/92, shown in bold). Two additional ODNs were selected to the left and right of the mutation. They were positioned in an asymmetrical manner with respect to the mutation and used as PCR primers to amplify an analytical DNA segment (598 bp): Exosc10ex10_2_Fwd 5′‐TCGCTATTACCATGTCGACCTCGAGtgaggaggcagatggttagc‐3′ and Exosc10ex10_2_Rev 5′‐CCTACCGGAATTCGCGTCTCGAGaccactgcctggctacattc‐3′. These primers also contained additional segments of DNA homology (shown in capital letters) to be used for Gibson‐cloning in bacteria and Sanger sequencing of the resulting mouse DNA fragments at subsequent steps, as previously described [68]. The selected sgRNA was functionally validated in an *in vitro* enzymatic cleavage assay with recombinant Cas9 protein (IDT) using the amplified PCR fragment as a target DNA sequence, resulting in two analytical bands of 425 bp and 173 bp. Fertilized mouse oocytes (1‐cell embryos, C57BL/6J) were electroporated with the crRNA+tracrRNA gRNA (200 ng/μl), the ssODN donor sequence (200 ng/μl), and recombinant Cas9 protein (IDT) (300 ng/μl) following procedures as described [69]. A total of 1859 1‐cell mouse embryos were electroporated in several independent sessions. Seven hundred and eighty‐eight mouse embryos having survived electroporation were implanted into 29 pseudopregnant females (CD1), resulting in nine F1 offspring that were further analyzed by Sanger DNA sequencing. In addition, 30 mouse blastocysts were individually analyzed after electroporation. All experimental procedures involving mice were validated by the local CNB Ethics Committee on Animal Experimentation. These procedures were then favorably evaluated by the institutional CSIC Ethics Committee and approved by the Autonomous Government of Madrid, in accordance with Spanish (RD53/2013) and European (Directive 2010/63/EU) legislation, with license number PROEX 009.4/21 to LM. All mice were housed at the registered CNB animal facility, where they had *ad libitum* access to food (regular rodent chow) and water. They were maintained on a light/dark cycle of 08:00–20:00. Both male and female mice were used indistinctly in the experiments. Mice used for the attempt to generate the animal models described in this paper were derived from original C57BL/6J mice obtained from Charles River Laboratories.

## 3 Results

### 3.1 EXOSC10 contains a conserved D‐box motif that includes a highly conserved serine at Position 402 mutated in colon cancer

**Fig. 1:**
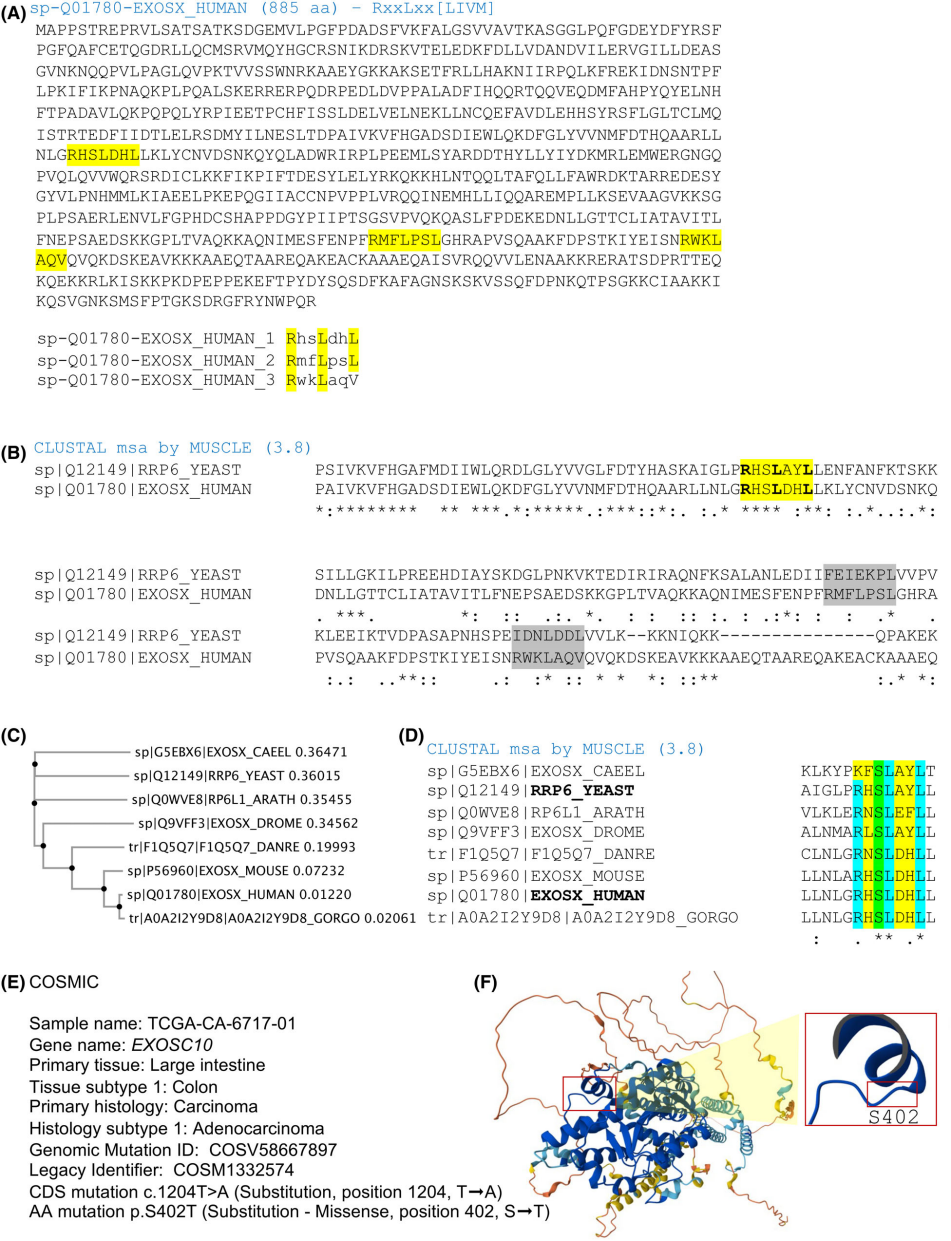
Scan for conserved D‐box and Catalog of Somatic Mutations in Cancer (COSMIC) mutations. (A) The human EXOSC10 protein sequence (UniProt Q01780) is shown. Three matches to the extended D‐box are highlighted in yellow (top). Aligned sequences of the matches are shown and conserved amino acids are marked in yellow (bottom). (B) A multiple sequence alignment (MSA) done with the EBI's MUSCLE software of yeast (UniProt Q12149) and human (UniProt Q01780) proteins is given. Conserved (*) and similar (:) amino acids are indicated at the bottom. (C) A phylogenetic tree of eight Rrp6/EXOSC10 orthologs created by the EBI's MUSCLE software is shown. (D) An MSA of D‐box matches in eight orthologs named via their UniProt unique identifiers is shown. Amino acids critical for D‐box function are highlighted in blue; S402 is highlighted in green; amino acids within the match are highlighted in yellow. (E) Data for EXOSC10 from COSMIC are given. (F) An EXOSC10 protein structure prediction from AlphaFold is shown (AF:Q01780). A red box marks the helix and the position of serine 402 (S402). An enlarged image of the helix is given and the position of S402 is indicated.

Given that the protein might be controlled under certain stress conditions via the proteasome pathway, we searched for sequence motifs known to mediate targeted proteolysis. The D‐box, recognized by the anaphase promoting complex/cyclosome (APC/C), has traditionally been defined by the minimal RxxL consensus. However, the Eucaryotic Linear Motif (ELM) resource suggests the motif RxxLxx[LIVM] better reflects the sequence conservation pattern observed in well‐studied substrates [57]. We employed scanprosite to analyze the amino acid sequence of human EXOSC10 and identified three matches that contained the core residues RxxLxxL/V (Fig. 1A) [52]. Next, we aligned the protein sequences of budding yeast Rrp6 and human EXOSC10 using multiple Sequence Comparison by Log‐Expectation (muscle) and found that the RHSLDHL motif was highly conserved (5/7 identical amino acids; Fig. 1B) [70]. Multiple sequence alignment of different species from primates to budding yeast revealed that the motif contains a highly conserved serine present even in the phylogenetically most remote species (budding yeast and worm, Fig. 1C,D).

We then searched the COSMIC database for mutations of any of the conserved amino acids of EXOSC10's D‐box match and found a missense mutation replacing serine 402 by threonine (S402T; Genomic Mutation ID: COSV58667897) that was detected in a colon‐cancer sample (Fig. 1E). Intriguingly, S402 is located within the catalytic domain of the human protein at an alpha helical structure as predicted by alphafold (AF:Q01780); we note that this prediction is based on a *bona fide* crystal structure (Fig. 1F) [61]. We considered it possible that S402 is directly (via the protein structure) or indirectly (via phosphorylation/dephosphorylation) important for the putative D‐box's function.

### 3.2 Mass spectrometry analysis of EXOSC10 and proteins that co‐purify with it reveals post‐translational modifications and interaction with RNA exosome co‐factors and APC/C subunits

**Fig. 2:**
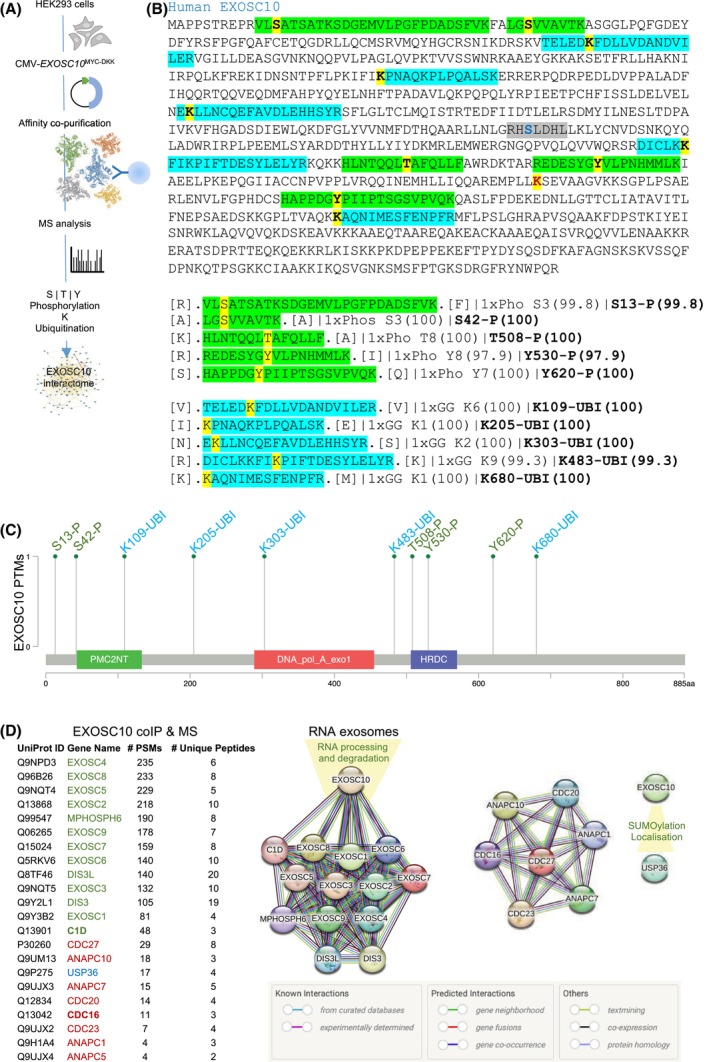
Mass spectrometry (MS)‐analysis of EXOSC10 and interacting proteins. (A) A schematic summarizes the experimental approach. The sample was injected twice (technical replicates *n* = 2). (B) The amino acid sequence of human EXOSC10 is shown at the top. Peptides containing phosphorylated and ubiquitinated residues are highlighted in green and blue, respectively. Modified amino acids are highlighted in yellow, the D‐box match is marked in light gray, S402 is shown in blue and K583 is shown in red. Peptide sequences and modifications are given at the bottom. (C) A lollipop plot shows post‐translational modifications (PTMs; y‐axis) within the protein sequence covering EXOSC10's N‐terminal protein interaction domain (NTD; PMC2NT), catalytic domain (CAT, EXO1, and HRDC) and C‐terminal domain (CTD; x‐axis). Modified amino acids are shown in green (phosphorylation) and blue (ubiquitination). (D) A table summarizes the output of selected proteins that co‐purified with EXOSC10. RNA exosome subunits are shown in green. Anaphase promoting complex/cyclosome (APC/C) subunits and co‐factors are shown in red. An enzyme that SUMOylates EXOSC10 is shown in blue. Identifiers are given as indicated at the top. The numbers (#) of peptide spectrum matches (PSMs) and unique peptides are shown. Protein network data from the String database are given for subunits of nuclear and cytoplasmic RNA exosomes, APC/C subunits and the EXOSC10 interactor USP36. Nodes and color‐coded edges are shown. A legend summarizes the color code of different types of interactions.

PhosphoSitePlus indicates EXOSC10 to be ubiquitinated at multiple residues and references S402 as a phosphorylated amino acid, but the latter evidence was obtained using the rat model organism [71]. We therefore sought to establish the pattern of post‐translational modifications (PTMs) of EXOSC10, with emphasis on ubiquitination and phosphorylation in this study, under normal growth conditions in cultured cells. To this end, we transfected HEK293T cells with a plasmid harboring a tagged human *EXOSC10* ^MYC‐DDK^ allele under the control of the CMV promoter and affinity purified the protein using Dynabeads coupled to an anti‐DDK antibody (Fig. 2A). The eluted protein was subjected to mass spectrometry analysis, which revealed that serine 13 (S13), serine 42 (S42), threonine 508 (T508), tyrosine 530 (Y530), and tyrosine 620 (Y620), but not S402, are phosphorylated under the culture conditions we employed (Fig. 2B). Furthermore, we found that lysines K109, K205, K303, K483, and K680 are ubiquitinated (Fig. 2B, File S1). These PTMs span almost the entire protein sequence and are localized within functional domains and the C‐terminal region (Fig. 2C). We conclude that EXOSC10 bears the ubiquitin PTM on five lysines in our experimental system, which is consistent with the presence of a highly conserved D‐box motif [72]. Moreover, among proteins that co‐purified under nondenaturing conditions with EXOSC10, we identified numerous known interactors. This includes the nuclear‐, nucleolar‐, and cytoplasmic RNA exosome core‐, and catalytic subunits (EXOSC1‐9, DIS3, DIS3L, and MPHOSPH6), the cofactor C1D (the mammalian ortholog of Rrp47 that controls the Rrp6 level) [73], the ubiquitin peptidase USP36, which mediates EXOSC10 SUMOylation [74] and seven subunits and co‐factors of the APC/C (Fig. 2D). The latter are present at very low concentrations, but they were unambiguously identified with at least two independent peptides (Fig. 2D, File S2).

### 3.3 S402 does not impair EXOSC10‐C1D interaction

**Fig. 3:**
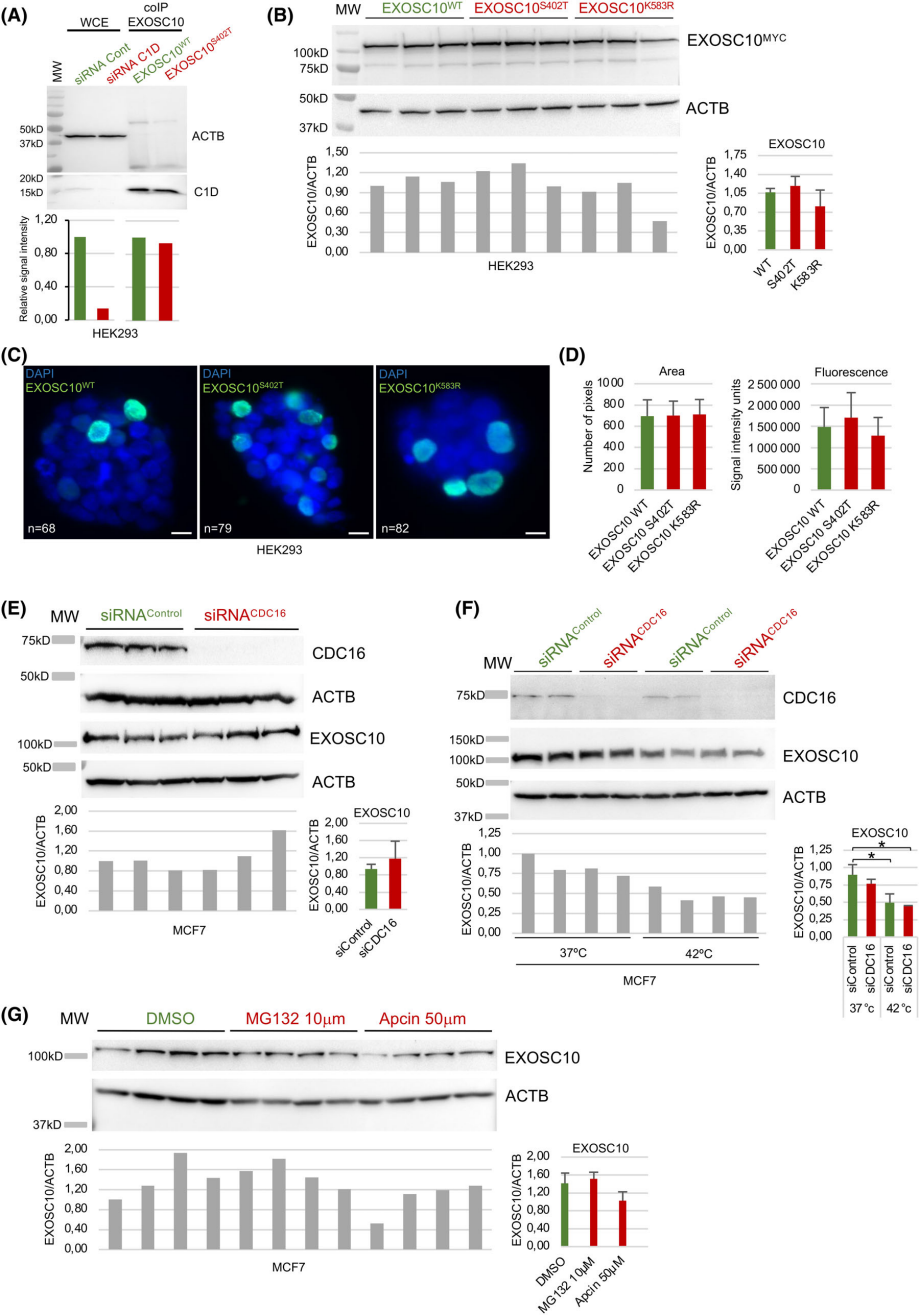
Molecular biological analysis of EXOSC10 stability under different conditions. (A) A western blot assay of whole cell extracts (WCE) and EXOSC10 co‐immunoprecipitated samples (Co‐IP) is shown (*n* = 1). Loading control (ACTB) and target protein (C1D) are indicated to the right. Relevant molecular weight markers (MW) are shown to the left. WCE samples from cells transfected with control siRNA (Cont, green) and target siRNA (C1D, red) are compared with Co‐IP samples from cells transfected with wild‐type (EXOSC10^WT^, green) and mutant (EXOSC10^S402T^, red) alleles as shown at the top. A color‐coded bar diagram at the bottom plots relative signal intensities (y‐axis) against samples as indicated (x‐axis). (B) A western blot of triplicate samples is shown (*n* = 3). Target and loading control proteins are indicated to the right; the target gene alleles are shown at the top and the cell line used is given at the bottom. Molecular weight markers (MW) are indicated. (C) Representative images of an immunocytochemistry assay revealing DNA (DAPI in blue) and proteins (Anti‐MYC) for tagged wild‐type (EXOSC10^WT^) and mutant (EXOSC10^S402T^, EXOSC10^K583R^) variants are shown in green as indicated in the top left corner. The number of cells analyzed for each allele (*n*) is given. Scale bar = 10 μm. (D) Color‐coded bar diagrams plot the number of pixels (left) or signal intensity units (right, y‐axis) against EXOSC10 wild‐type (green) and mutant (red) alleles (x‐axis). The error bars indicate standard deviation. (E) A western blot is shown like in panel B. A bar diagram to the left plots normalized intensity units (EXOSC10/ACTB; y‐axis) against triplicate samples (x‐axis; *n* = 3). A bar diagram to the right plots averaged intensity units (y‐axis) against control and experimental samples (x‐axis). The error bars indicate standard deviation. The target protein is given at the top. (F–G) Western blots, quantified band intensity signals, and averaged signals are shown like in panel B for representative duplicate (panel F, total n = 4; anova followed by Tukey's post hoc range test, Lanes 1 versus 3: *p* = 0.05 and Lanes 1 versus 4: *p* = 0.04; * = significant) and quadruplicate (panel G, *n* = 4) samples.

We sought to validate EXOSC10 interactors identified by mass spectrometric analysis, and address the possibility that S402T, although located within the catalytic domain, affects protein–protein interactions indirectly via structural alterations that reduce or impair its protein‐binding capacity. To this end, we chose the critical interactor C1D, the yeast ortholog of which is needed to stabilize the EXOSC10 ortholog Rrp6. We first confirmed that the antibody specifically detects the intended target by assaying protein extracts from cells transfected with control siRNA and C1D siRNA (Fig. 3A). We then monitored C1D in Co‐IP samples from cells transfected with tagged EXOSC10 wild‐type and S402T alleles and found that the bands representing C1D showed highly similar intensities (Fig. 3A). This result confirms the Co‐IP/mass spectrometry data shown in Fig. 2D, and suggests that EXOSC10^S402T^ binds C1D under optimal growth conditions as efficiently as the wild‐type allele. While we cannot categorically rule out that other EXOSC10‐interacting proteins are affected by S402T, we find it unlikely, given the position of S402T and unperturbed C1D binding.

### 3.4 S402 is stable and shows the same localization pattern as the wild‐type protein

Next, we sought to determine the effect of S402T on protein stability, and subcellular localization during normal mitotic cell cycle progression. We compared the allele to K583R as a negative control, because K583 was recently shown to be deSUMOylated by USP36 in response to limited oxygen supply (hypoxia), a process that triggers EXOSC10's translocation from the nucleolus to the nucleus but does not appear to affect the protein's stability [75]. A western blot assay revealed no difference in the levels of CMV promoter‐driven tagged wild‐type, S402T and K583R alleles (anova, *p* = 0.149; Fig. 3B). Furthermore, we quantitatively assayed tagged wild‐type EXOSC10 and the S402T and K583R variants in efficiently transfectable HEK293T cells using an anti‐MYC antibody, and found that they show similar nuclear staining patterns (Fig. 3C,D). Taken together, the results suggest that S402 is not involved in regulating EXOSC10 protein stability or subcellular localization under optimal growth conditions.

### 3.5 S402 and the APC/C are not required for EXOSC10 stability during mitotic cell growth and division

Subsequently, we asked whether the APC/C‐proteasome pathway controls EXOSC10 levels. Depleting the essential core subunit CDC16|APC6, which is required for the APC/C's integrity, caused no significant increase of EXOSC10's level under normal growth conditions (Student *t*‐test *p* = 0.4; Fig. 3E) [76, 77]. We repeated the experiment comparing standard temperature (37 °C) and heat shock (42 °C). We found that heat stress reduces EXOSC10; however, this appears to occur independently of CDC16|APC6 (Figure F) and compare samples in Lanes 1 versus 3 (Tukey *p* = 0.05) and Lanes 1 versus 4 (Tukey *p* = 0.04). Finally, we observed no significant effect on EXOSC10 steady‐state levels after treatment with an APC/C inhibitor (Apcin 50 μm) and a proteasome inhibitor (MG132 10 μm; anova *p* = 0.150; Fig. 3G). These results argue against a role for D‐box mediated proteolysis of EXOSC10 by the APC/C during mitotic growth and in response to temperature stress, and suggest that the S402T mutation does not affect protein stability to a detectable level under the conditions we have investigated.

### 3.6 The prevalence of EXOSC10 LoF alleles in the human population

**Fig. 4:**
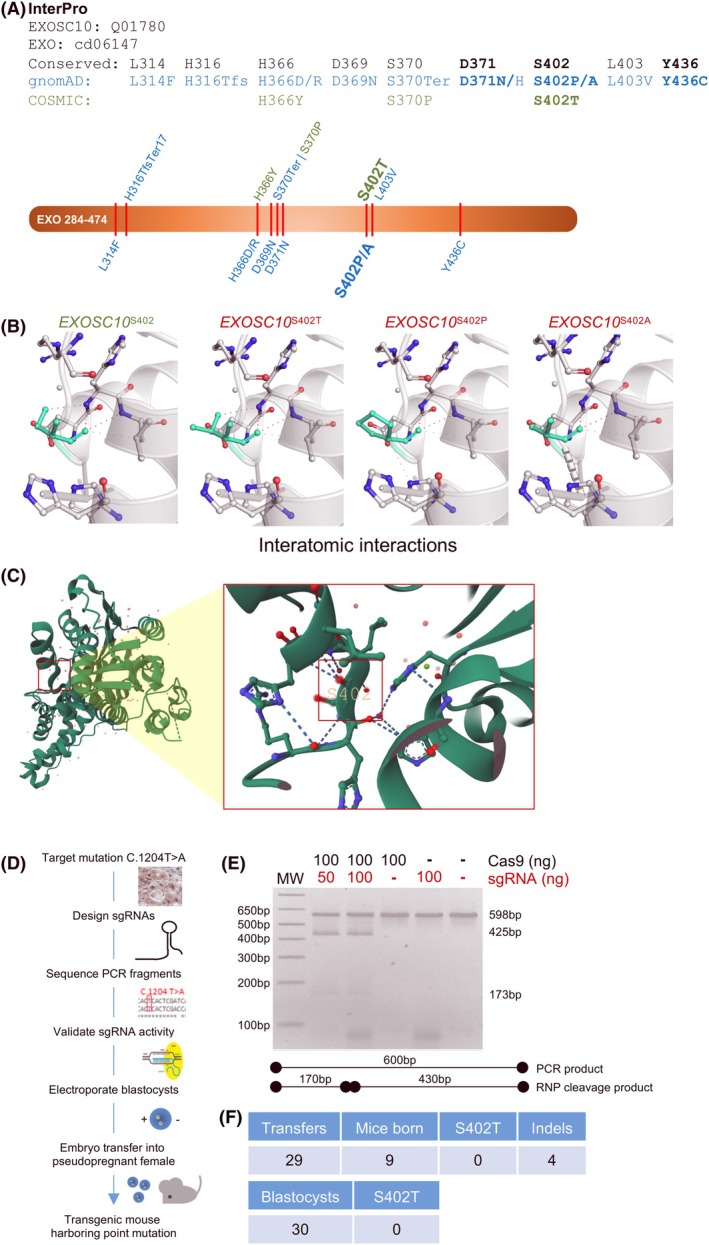
S402 position and atomic interactions within EXOSC10 and phenotypic analysis of *Exosc10* ^S402T^. (A) A schematic shows the positions of conserved amino acids (interpro) and the corresponding missense mutations identified in the human population (gnomad). (B) Atomic interactions of serine, threonine, alanine, and proline at Position 402 created using dynamut are shown. The target amino acid is given in light green. Dotted lines symbolize interactions. (C) The structure of EXOSC10's catalytic subunit provided by Protein Databank is shown. A red box marks the position of S402. An enlarged image shows the interactions of S402 with neighboring residues. (D) A flow chart shows the experimental approach to generating a transgenic gene deletion model. (E) A gel displays the outcome of a cleavage assay. Lane 1 contains molecular weight markers (MW); Lanes 2–6 contain different amounts in nanograms (ng) of sgRNA and Cas9 protein as indicated at the top. The sizes of DNA fragments are shown in base pairs (bp) to the left. A schematic at the bottom represents the target DNA and cleavage fragments. (F) Data showing the outcome of the gene editing experiment are summarized for postnatal (top) and embryonic (bottom) samples as indicated.

InterPro provides information on highly conserved amino acids in EXOSC10, including six cases referenced as mutated in gnomAD and three cases referenced both in gnomAD and COSMIC (Fig. 4A, Table 2). Data from gnomAD indicate the presence of heterozygous *EXOSC10* ^S402P^ (1‐11 082 764‐A‐G) and *EXOSC10* ^S402A^ (1‐11 082 764‐A‐C) variants in the human genome, albeit at a low allelic frequency (6.2 × 10^−7^). To assess their phenotypic potential, we employed PolyPhen‐2 and found that S402P [*HumDiv* score of 1.000 (sensitivity: 0.00; specificity: 1.00); *HumVar* score 0.999 (sensitivity: 0.09; specificity: 1.00)] and S402A [*HumDiv* score of 0.999 (sensitivity: 0.14; specificity: 0.99); *HumVar* score 0.998 (sensitivity: 0.18; specificity: 0.98)] alleles are *probably damaging* [54]. Moreover, AlphaMissense classifies S402P as *likely pathogenic* (Score 0.9937) and S402A as *ambiguous* (Score 0.5227) [55].

**Table 2:** Mutations of InterPro's highly conserved amino acids in *EXOSC10*. gnomAD identifiers, the effect at the protein level, the allele count (AC), and the allele frequency (AF) are indicated.

gnomAD ID	Protein	AC	AF
1‐11 082 857‐C‐G	p.Asp371His	7	0.000004336572546522131
1‐11 082 871‐T‐C	p.His366Arg	6	0.000003717596499511136
1‐11 081 212‐T‐C	p.Tyr436Cys	4	0.0000024780445255040344
1‐11 087 565‐TCCCAGGAAGCTCCTGTAAGAGTGGTG‐T	p.His316ThrfsTer17	3	0.0000018588235877277989
1‐11 082 859‐G‐C	p.Ser370Ter	2	0.0000012390483612965898
1‐11 082 764‐A‐G	p.Ser402Pro	1	6.195042231602893e‐7
1‐11 082 764‐A‐C	p.Ser402Ala	1	6.195042231602893e‐7
1‐11 082 857‐C‐T	p.Asp371Asn	1	6.195103637888758e‐7
1‐11 082 863‐C‐T	p.Asp369Asn	1	6.195211101818295e‐7
1‐11 082 872‐G‐C	p.His366Asp	1	6.196001843930149e‐7
1‐11 087 803‐C‐G	p.Leu314Phe	1	6.208272647468204e‐7

Next, we investigated the impact of S402T and S402P mutations on protein dynamics and stability that stem from vibrational entropy changes using dynamut and found that both are predicted to have a stabilizing effect but to decrease the mutant protein's flexibility (S402T: stability ΔΔG: 0.672 kcal·mol^−1^, flexibility ΔΔS_Vib_ ENCoM: −0.095 kcal.mol^−1^·K^−1^; S402P: stability ΔΔG 0.718 kcal·mol^−1^, flexibility ΔΔS_Vib_ ENCoM: −0.123 kcal.mol^−1^·K^−1^) [56]. Unsurprisingly, the S402P mutation alters interatomic interactions (Fig. 4B).

The dynamut prediction is concordant, as far as protein stability is concerned, with our biochemical analysis of *EXOSC10* wild‐type versus S402T and K583R mutant alleles in cultured cells (see Fig. 3A). It is conceivable that a more rigid structure within the catalytic domain may render S402T and S402P nonfunctional.

### 3.7 Exosc10S402T causes an early embryonic lethal phenotype

We hypothesized that the *EXOSC10* ^S402T^ colon‐cancer allele may display altered stability or function and thereby exert a clinically relevant effect in tumor tissue. After having accumulated evidence arguing against a role in protein stability and subcellular localization, we sought to assay the allele's function *in vivo* given that S402 is part of a highly conserved sequence motif located within the catalytic EXO1 domain and that S402T is predicted to be a damaging mutation (Fig. 4C). We employed a CRISPR/Cas9‐based editing method that involves electroporation of small guide RNAs (sgRNAs) within a riboprotein complex into blastocysts (Fig. 4D and Fig. S1). The sgRNA performed as expected when combined with Cas9 *in vitro* (Fig. 4E). A total of 29 electroporation samples were transferred into females, but among nine mice, none bore the S402T mutation, while four mice bore small deletions (Fig. 4F and Fig. S2). Next, we analyzed another 30 electroporated blastocysts and found that none bore the S402T allele.

These data imply that *Exosc10* ^*S402T*^ is incompatible with mouse blastomere growth and division, which is consistent with our previous findings using an *Exosc10* global gene deletion model [34]. It is unlikely that S402T is a dominant negative allele given that it was detected in colon‐cancer cells. Collectively, our results suggest that S402T—and by inference, S402P and S402A—are recessive LoF alleles that may influence 5‐FU sensitivity in tumors and heterozygous carriers.

## 4 Discussion

### 4.1 The role of post‐translational modifications in regulating EXOSC10's stability and localization

EXOSC10 undergoes a variety of PTMs, including ubiquitination and sumoylation, whereby the latter has been associated with temperature‐dependent instability and translocation of the protein from the nucleolus into the nucleoplasm in response to oxygen depletion [12, 74, 75]; reviewed in [17]. In earlier work, we found that yeast Rrp6 levels progressively declined when cells switched from fermentation (rapid mitotic growth in the presence of glucose) to respiration (mitotic growth with acetate as a sole carbon source) and sporulation (meiotic differentiation enabling a diploid yeast cell to form four haploid spores in response to nutritional cues), while the mRNA remained detectable [15]. In an analogous experiment, we observed a similar decline of the rodent EXOSC10 protein (but not mRNA) when mitotic spermatogonia develop into meiotic spermatocytes and postmeiotic round spermatids (Fig. S3A [78], see Fig. S2A–C in reference [14]). Moreover, we find that FBS starvation (at 2% FBS versus the standard 8%) decreases the EXOSC10 protein level, while it induces the mRNA (Supplemental Fig. S3B,C). Such observations suggest that EXOSC10 is regulated predominantly at the post‐translational level in response to nutritional stress conditions; for review, see [17].

A recent functional genomics analysis of human protein domains revealed that approximately 60% of pathogenic missense mutations reduce protein stability [79]. We hypothesized that the highly conserved S402 within a conserved D‐box motif in mammalian EXOSC10 might be involved in abnormal cancer cell growth by altering the protein's stability or function. We find, however, that *EXOSC10* ^S402T^ shows no discernible effect on protein levels and subcellular localization when expressed under the CMV promoter in cultured HEK293 cells. It could be that S402 is not relevant for the D‐box's function or that critical arginine and leucine residues within the conserved motif or even all three matches would have to be mutated to observe an effect. We consider this unlikely, however, given that depleting cells of the APC/C subunit CDC16|APC6 and treating them with APC/C and proteasome inhibitors did not increase EXOSC10 levels under optimal growth conditions and during heat shock [19]. The presence of APC/C subunits among the set of proteins co‐purified with EXOSC10 might be explained by their interaction with EXOSC10‐associated factors. Indeed, mass spectrometry‐based detection of proteins that bind the APC/C adaptor FZR1|CDH1 identified seven out of nine RNA exosome core subunits (see File S2 of reference [80] and www.thebiogrid.org).

Taken together, the data argue against a critical role for the APC/C in regulating EXOSC10's stability during mitotic cell growth and heat stress response. It will be interesting to further explore proteolytic pathways to decipher the roles of EXOSC10's ubiquitin code, and the mechanisms underlying its external cue‐induced instability under various growth conditions and in developmental pathways.

### 4.2 Heterozygous EXOSC10 LoF alleles in tumors and 5‐FU sensitivity

We find that S402T causes an early embryonic lethal phenotype in a transgenic mouse model akin to the effect of a gene deletion experiment [34]. Given that S402T cannot be a dominant negative allele due to its presence in a colon‐cancer sample, the simplest interpretation of our results is that *EXOSC10* ^S402T^ is a LoF allele. We note that *Exosc10* ^*+/−*^ heterozygous mutant mice display no apparent phenotype, which suggests that 50% of EXOSC10's physiological level (hence activity) is sufficient for normal pre‐ and postnatal cell growth and development, at least under laboratory conditions [34]. However, earlier work showed that reducing human *EXOSC10* several‐fold in cultured HEp‐2 cancer cells via siRNA partially impairs mitotic cell cycle progression, which indicates that there is a threshold level of the enzyme critical for normal cell growth and division [81]. Importantly, low levels of EXOSC10 correlate with increased 5‐FU sensitivity in cultured cells, and a recent study reveals that a mechanistic link underlies this observation [47]: a major evolutionarily conserved role of Rrp6/EXOSC10 is to process ribosomal RNA (rRNA), which is inhibited by incorporation of fluorinated uracil into its substrates [9, 22, 47, 48]. The importance of altered EXOSC10 activity in the context of 5‐FU's cytotoxic effects is underlined by work showing that the drug is incorporated into rRNA and mostly acts via a rRNA damage response pathway, leading to impaired ribosome biogenesis [45]. This suggests a direct connection between 5‐FU's major mechanism of action and EXOSC10's cellular concentration and activity.

Transmission of recessive *EXOSC10* LoF alleles via the germline must be distinguished from *de novo* creation of such alleles in tumors because the former precludes standard 5‐FU treatment, while the latter suggests tumoral drug hypersensitivity, which may allow for adjusting 5‐FU concentrations to help alleviate secondary effects. Critically, among *EXOSC10* missense mutants lacking enzymatic activity one possibly equivalent allele is referenced in COSMIC (E315Q: E315K/COSM9280699), and one identical and two conceivably equivalent heterozygous missense mutations are referenced in gnomAD [D371N: D371N/1‐11 082 857‐C‐T (allele frequency 6.2 × 10^−7^), D371H/1‐11 082 857‐C‐G (4.3 × 10^−6^); Y436A: Y436C/1‐11 081 212‐T‐C (2.5 × 10^−6^)] (Fig. S4A) [38]. This reveals the presence of rare but potentially clinically relevant missense mutations that are known to be (or that very likely constitute) LoF alleles in tumors and the human population. In this context, it is noteworthy that querying a set of 10 967 cancer samples from 10 953 patients at cBioPortal (https://www.cbioportal.org; tcga_pan_can_atlas_2018) reveals that 1281 (12%) bore altered *EXOSC10* alleles, whereby endometrial, bladder, skin, colorectal and cervical cancers are among those that harbor the greatest number of mutations (Fig. S4B; File S3) [58]. This is a rich source of data for further exploration and functional assays, given that the presence of *EXOSC10* ^S402T^ or equivalent LoF alleles could be an indicator of a potentially favorable 5‐FU‐based chemotherapeutical outcome.

### 4.3 Heterozygous EXOSC10 LoF alleles in carriers and 5‐FU toxicity

At the organismic level in humans, heterozygous deleterious termination, frameshift and experimentally verified missense *EXOSC10* LoF alleles transmitted through the germline could mediate a rare but potentially fatal 5‐FU hypersensitivity phenotype. The functional consequences of missense mutations that replace any given amino acid by another that is more or less similar in structure and charge are difficult to predict. Typically, evolutionary conservation, PTMs and positions within critical domains are considered to be useful criteria for assessing a residue's functional relevance. Given that *EXOSC10* is essential but not haploinsufficient (i.e., heterozygous *Exosc10* mutant mice display no discernible phenotype), homozygous mutations should in all likelihood not be LoF alleles [34]. It is consistent with this assumption that all 24 homozygous single nucleotide variants (SNVs) referenced in gnomAD for *EXOSC10* are missense mutations located outside of the catalytic domains, except N301S in EXO1 and E568K in HRDC (Fig. S5A top, File S4). As one would expect, N301 and E568 are not conserved (Fig. S5A bottom). Furthermore, according to data from gnomAD, N301S (SIFT score 0.240 and PolyPhen score 0.262) and E568K (SIFT score 0.190 and PolyPhen score 0.263) are not predicted to be *probably damaging*. Moreover, alphamissense, a powerful prediction algorithm taking protein structure and evolutionary conservation into account, classifies them as benign (N301S: score 0.0954, class *likely benign*; E568K: 0.281, *likely benign*) [54, 55]. The data, together with the notable absence of homozygous frameshift or premature termination mutants, suggest that the catalytic domain of human EXOSC10 (comprising EXO1 and HRDC) does not harbor homozygous LoF alleles.

Conversely, any mutations that remove or alter the catalytic domains almost certainly yield inactive proteins. gnomAD references 74 termination (ter) and frameshift (fs) mutants with a total allele count of 240 and a combined allele frequency of 1.5 × 10^−4^ that encode truncated proteins from Position 1 to 543 (the mutation at Position 543 was selected because it removes half of the HRDC domain; supplemental Fig. S5B and File S5). Among cases showing at least five allele counts, four heterozygous mutations referenced by gnomAD creating stop codons (K159Ter/1‐11 091 495‐T‐TA, R182Ter/1‐11 091 113‐G‐A, Y260Ter/1‐11 088 177‐A‐T and Y319Ter/1‐11 087 580‐G‐T) and four alleles causing frameshifts (S42IfsTer10, E299NfsTer7/1‐11 087 849‐TC‐T, Y410LfsTer13/1‐11 082 739‐TAG‐T, M536DfsTer6/1‐11 080 528‐CAT‐C) yield truncated proteins that lack most or all of the catalytic EXO1 and HRDC domains and therefore most likely represent LoF alleles (Fig. S5B, Tables 3 and 4). The currently known pool of ter/fs alleles represents a minimal set since additional mutations within the C‐terminal region of the HRDC domain, and alterations within the unstructured C terminus of EXOSC10 may yield unstable mutant proteins or alleles that fail to interact normally with their co‐factors.

**Table 3:** *EXOSC10* mutations creating stop codons. gnomAD identifiers, the effect at the protein level, the allele count (AC), and allele frequency (AF) are indicated for the top 10 most frequent alleles.

gnomAD ID	Protein	AC	AF
1‐11 088 177‐A‐T	p.Tyr260Ter	43	0.000026658367441577878
1‐11 087 580‐G‐T	p.Tyr319Ter	23	0.000014250115549850002
1‐11 068 066‐T‐A	p.Lys857Ter	11	0.000006814681549931171
1‐11 069 703‐G‐A	p.Arg782Ter	11	0.000006816826903412999
1‐11 066 736‐G‐T	p.Tyr880Ter	10	0.000006194934773531769
1‐11 091 113‐G‐A	p.Arg182Ter	7	0.000004337088425797993
1‐11 091 495‐T‐TA	p.Lys159Ter	7	0.0000043377065677834815
1‐11 069 655‐G‐A	p.Arg798Ter	6	0.0000037171911416856966
1‐11 079 731‐G‐A	p.Arg577Ter	6	0.000003718527314442388
1‐11 069 673‐G‐A	p.Gln792Ter	5	0.00000310205641523879
1‐11 070 914‐G‐A	p.Arg768Ter	5	0.0000030979049488411975

**Table 4:** *EXOSC10* mutations creating frameshifts. gnomAD identifiers, the missense mutation, and the allele count (AC) and allele frequency (AF) are indicated for the top 10 most frequent alleles.

gnomAD ID	Protein	AC	AF
1‐11 098 122‐GCCTTGGTGACTGCCACCACGGAC‐G	p.Ser42IlefsTer10	38	0.000023578700739130218
1‐11 069 664‐CTT‐C	p.Gln794ArgfsTer47	17	0.000010536689372199255
1‐11 069 657‐TTCTTC‐T	p.Lys796ThrfsTer44	12	0.00000745162960929864
1‐11 072 109‐CT‐C	p.Lys740ArgfsTer34	12	0.000007435911735727697
1‐11 080 528‐CAT‐C	p.Met536AspfsTer6	8	0.000005004028242735402
1‐11 076 844‐TA‐T	p.Phe661LeufsTer14	6	0.000003724177484367765
1‐11 082 739‐TAG‐T	p.Tyr410LeufsTer13	6	0.0000037170207335416516
1‐11 069 607‐CTT‐C	p.Lys813ArgfsTer28	5	0.0000030975172779513763
1‐11 087 849‐TC‐T	p.Glu299AsnfsTer7	5	0.0000030977782734223015

Contrary to mutations that yield truncated proteins lacking essential domains, the effect of most missense mutations is challenging to predict but the methods have been improving steadily. The state‐of‐the‐art AlphaMissense algorithm scores 1617 out of 5866 *EXOSC10* missense mutations (28%) as *likely pathogenic* [55]. Among 56 missense mutations detected in 521 samples (combined allele frequency 3.2 × 10^−4^) located within the PMC2NT, EXO1 and HRDC domains and possessing SIFT and PolyPhen scores of 0 and 1, respectively, all but four likely benign cases are confirmed by AlphaMissense as *likely pathogenic* (47 cases) or *ambiguous* (five cases; Fig. S5C,D and File S6). This further increases the pool of alleles that are potentially clinically relevant. Collectively, our results suggest *EXOSC10* to be a 5‐FU sensitivity biomarker. As such, it might be a useful complement of thymidine synthase (*TYMS*), thymidine phosphorylase (*TYMP*) and dihydropyrimidine dehydrogenase (*DPYD*), for which mutant alleles can explain many but not all cases of fluoropyrimidine toxicity; reviewed in [82] (see Tables 1, 2, 3 and Fig. 4E).

## 5 Conclusions

EXOSC10 undergoes a variety of PTMs, and acts as a hub protein within an extensive protein–protein interaction network. Our findings suggest that the colon‐cancer allele EXOSC*10* ^S402T^ does not affect protein stability and subcellular localization but *in vivo* function. Given that decreased EXOSC10 levels enhance 5‐FU toxicity, the results are potentially relevant for drug treatment of tumors with heterozygous *EXOSC10* LoF alleles and for cancer patients carrying rare but critical mutations when undergoing 5‐FU‐based chemotherapy. The present study is of an exploratory nature, and paves the way for more exhaustive work that includes additional types of mutations, such as insertions and deletions, mutations that affect mRNA splicing or alterations that impair 5′‐ and 3′‐UTR function in cancers and the human population. Further studies are also needed to investigate the regulation of EXOSC10's stability and subcellular localization during cell growth and physical, chemical and nutritional stress responses in normal and cancer cells. Finally, our results set the stage for analyzing EXOSC10's PTMs (including methylation, acetylation, phosphorylation, sumoylation and ubiquitination) that are altered by 5‐FU treatment or serum starvation, and that involve amino acids, which are mutated in somatic cancers and heterozygous carriers.

## Conflict of interest

The authors declare no conflict of interest.

## Author contributions

RS, YLP, and FP performed experiments. EB analyzed genomics data. LN analyzed mass spectrometry data. AF, MC, DMS, and LM generated transgenic mice. CG contributed to experimental design. MP contributed to experimental design, analyzed and interpreted data, and wrote the manuscript. All authors contributed to the manuscript.

## Supporting information


**Fig. S1.** CRISPR/Cas9 primer and sgRNA sequences.

**Fig. S2.** CRISPR/Cas9 generated mutants in F1 mice.

**Fig. S3.** EXOSC10 stability during nutritional stress.

**Fig. S4.** cBioPortal data for *EXOSC10*.

**Fig. S5.** gnomAD data for *EXOSC10*.

**File S1.** Mass spectroscopy data for EXOSC10.

**File S2.** Mass spectroscopy data for EXOSC10 co‐purified proteins.

**File S3.** sBioPortal data for *EXOSC10*.

**File S4.** gnomAD data for missense mutations in *EXOSC10*.

**File S5.** gnomAD data for frameshift and termination mutations in *EXOSC10*.

**File S6.** gnomAD data for missense mutations in *EXOSC10 domains*.

## Data Availability

Supporting data are available as Supplementary information. The mass spectrometry proteomics data have been deposited in the ProteomeXchange Consortium (https://proteomecentral.proteomexchange.org) via the Proteomics Identifications Database (PRIDE) repository (https://www.ebi.ac.uk/pride/) with the dataset identifier PXD061578.
