# Supplementary material for: Heterozygous loss‐of‐function alleles associate the conserved 3′‐5′ exoribonuclease EXOSC10 with hypersensitivity to the anticancer drug 5‐fluorouracil
Source: Mol Oncol. 2026 May 15;20(8):1913–32. doi: 10.1002/1878-0261.70239 (PMC13398982; doi:10.1002/1878-0261.70239)
Supplement: Supplementary file 1 [file MOL2-20-1913-s002.pdf]

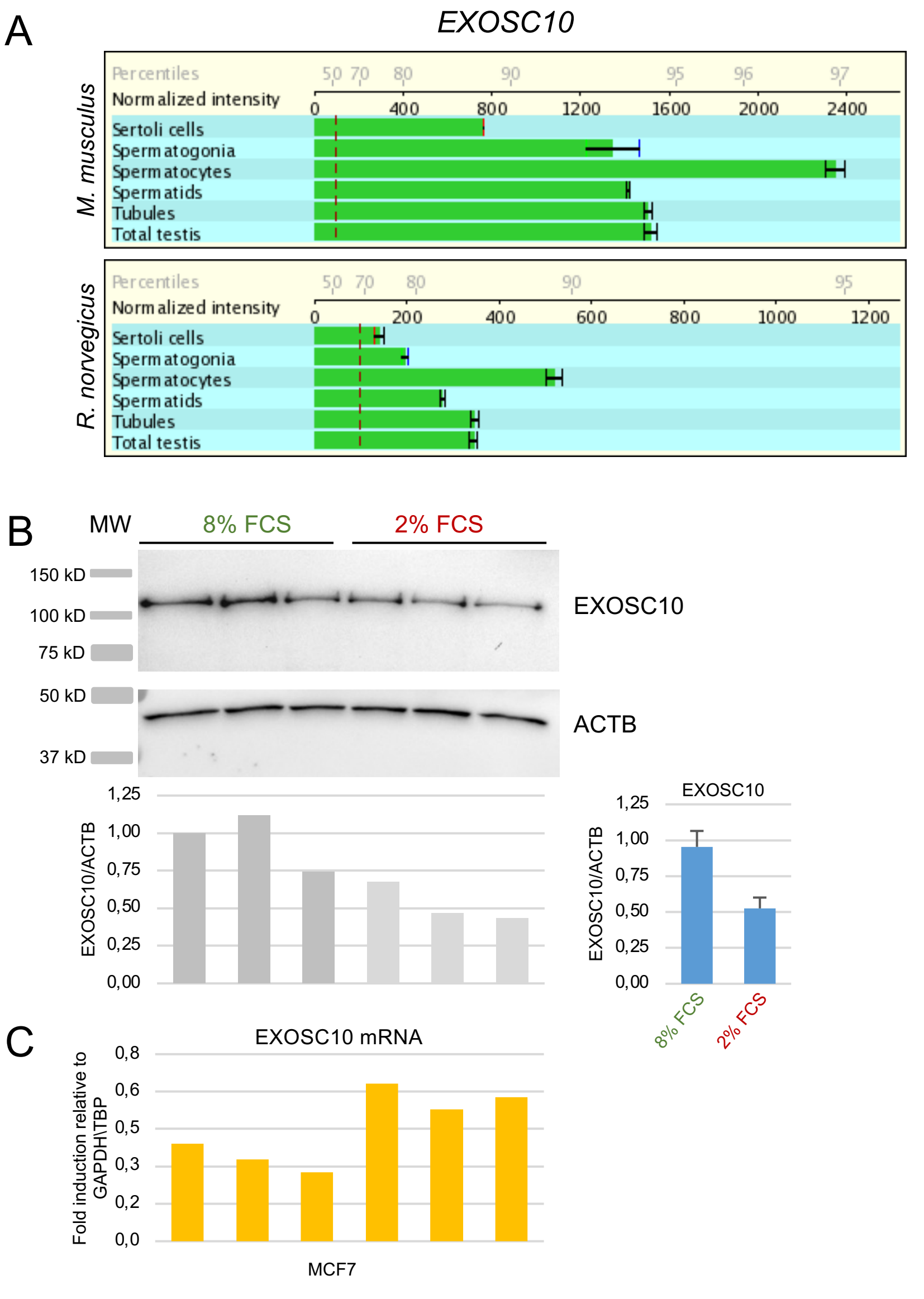

Supplemental Figure S3. EXOSC10 stability during nutritional stress. (A) Bar diagrams plot enriched cell populations and total testicular samples from rodents as indicated to the left (y-axis) against *EXOSC10* mRNA levels (normalized intensity signals, x-axis). The standard deviation and percentiles are indicated. The data were reported by Chalmel *et al*., Proc Natl Acad Sci, USA 2007; the graphs were retrieved from GermOnline. (B) A Western blot and protein quantification data are shown for triplicate samples from MCF7 cells harvested under conditions using different concentrations of fetal calf serum (FCS) given at the top (n=3). The target protein and the loading control are shown to the right; their molecular weight is given in kilo Dalton (kD). The bar diagram to the left plots samples (x-axis) against relative signal intensities for EXOSC10 protein normalized over ACTB (y-axis). The bar diagram to the right plots averaged samples cultured in the presence of high (green) and low (red) FCS concentrations (x-axis) against normalized averaged signal intensities (y-axis). The error bars indicate standard deviation. Molecular weight (MW) markers are indicated in kilo Dalton (kD). (C) A bar diagram plots triplicate samples for normal (8%) and FCS starvation (2%) conditions (x-axis) against the fold-change of normalized expression signals for the target gene shown at the top against averaged signals obtained for two housekeeping genes (*GAPDH*, *TBP*).
