# Supplementary material for: Heterozygous loss‐of‐function alleles associate the conserved 3′‐5′ exoribonuclease EXOSC10 with hypersensitivity to the anticancer drug 5‐fluorouracil
Source: Mol Oncol. 2026 May 15;20(8):1913–32. doi: 10.1002/1878-0261.70239 (PMC13398982; doi:10.1002/1878-0261.70239)
Supplement: Supplementary file 2 [file MOL2-20-1913-s006.pdf]

Exosc10 exon10 wt

```
 M   F   D   T   H   Q   A   A   R   L   L   N   L   A   R   H   S   L   D   H   L   L   R   L
…ATG TTT GAC ACA CAC CAG GCA GCA CGG CTT CTC AAC CTG GCT CGG CAC TCA CTC GAC CAT CTG CTG AGA CTC
TAC TGC GGT GTG GAA TCA AAC AAG CAA TAT CAG CTG GCA GAC TGG AGG ATA CG…
 Y   C   G   V   E   S   N   K   Q   Y   Q   L   A   D   W   R   I
```

Exosc10 exon10 mutS402T

```
 M   F   D   T   H   Q   A   A   R   L   L   N   L   A   R   H   T   L   D   H   L   L   R   L
…ATG TTT GAC ACA CAC CAG GCA GCA CGG CTT CTC AAC CTG GCT CGG CAC ACA CTC GAC CAT CTG CTG AGA CTC
TAC TGC GGT GTG GAA TCA AAC AAG CAA TAT CAG CTG GCA GAC TGG AGG ATA CG…
 Y   C   G   V   E   S   N   K   Q   Y   Q   L   A   D   W   R   I
```

Exosc10 exon10 F0 2 (03/04/18) ♂ fs42*

```
 M   F   D   T   H   Q   A   A   R   L   L   S            S   A   L   T   R   P   S   A   E   T
…ATG TTT GAC ACA CAC CAG GCA GCA CGG CTT CTC A-- --- GC  TCG GCA CTC ACT CGA CCA TCT GCT GAG ACT
```

Exosc10 exon10 F0 4 (03/04/18) ♂ fs8*

```
 M   F   D   T   H   Q   A   A   R   L   L   N   L            G   T   H   S   T   I   C   *
…ATG TTT GAC ACA CAC CAG GCA GCA CGG CTT CTC AAC CT- --- C GGC ACT CAC TCG ACC ATC TGC TGA
```

Exosc10 exon10 F0 5 (03/04/18) ♂ fs8*

```
 M   F   D                                                  G     T   H   S   T   I   C   *
…ATG TTT GAC --- --- --- --- --- --- --- --- --- --- --- -GG C ACT CAC TCG ACC ATC TGC TGA
```

Exosc10 exon10 F0 6 (03/04/18) ♀ fs10*

```
 M   F   D   T   H   Q   A   A   R   L   L   L           L   G   T   H   S   T   I   C   *
…ATG TTT GAC ACA CAC CAG GCA GCA CGG CTT CTT TT- --- G  CTC GGC ACT CAC TCG ACC ATC TGC TGA
```

Supplemental Figure S2. CRISPR/Cas9 generated mutants in F1 mice. The wild-type and S402 mutant DNA (black) and protein (blue) sequences are compared to four frameshift (fs) mutations observed in three males and one female. The target base is shown in green (T).
