# Supplementary material for: Heterozygous loss‐of‐function alleles associate the conserved 3′‐5′ exoribonuclease EXOSC10 with hypersensitivity to the anticancer drug 5‐fluorouracil
Source: Mol Oncol. 2026 May 15;20(8):1913–32. doi: 10.1002/1878-0261.70239 (PMC13398982; doi:10.1002/1878-0261.70239)
Supplement: Supplementary file 3 [file MOL2-20-1913-s007.pdf]

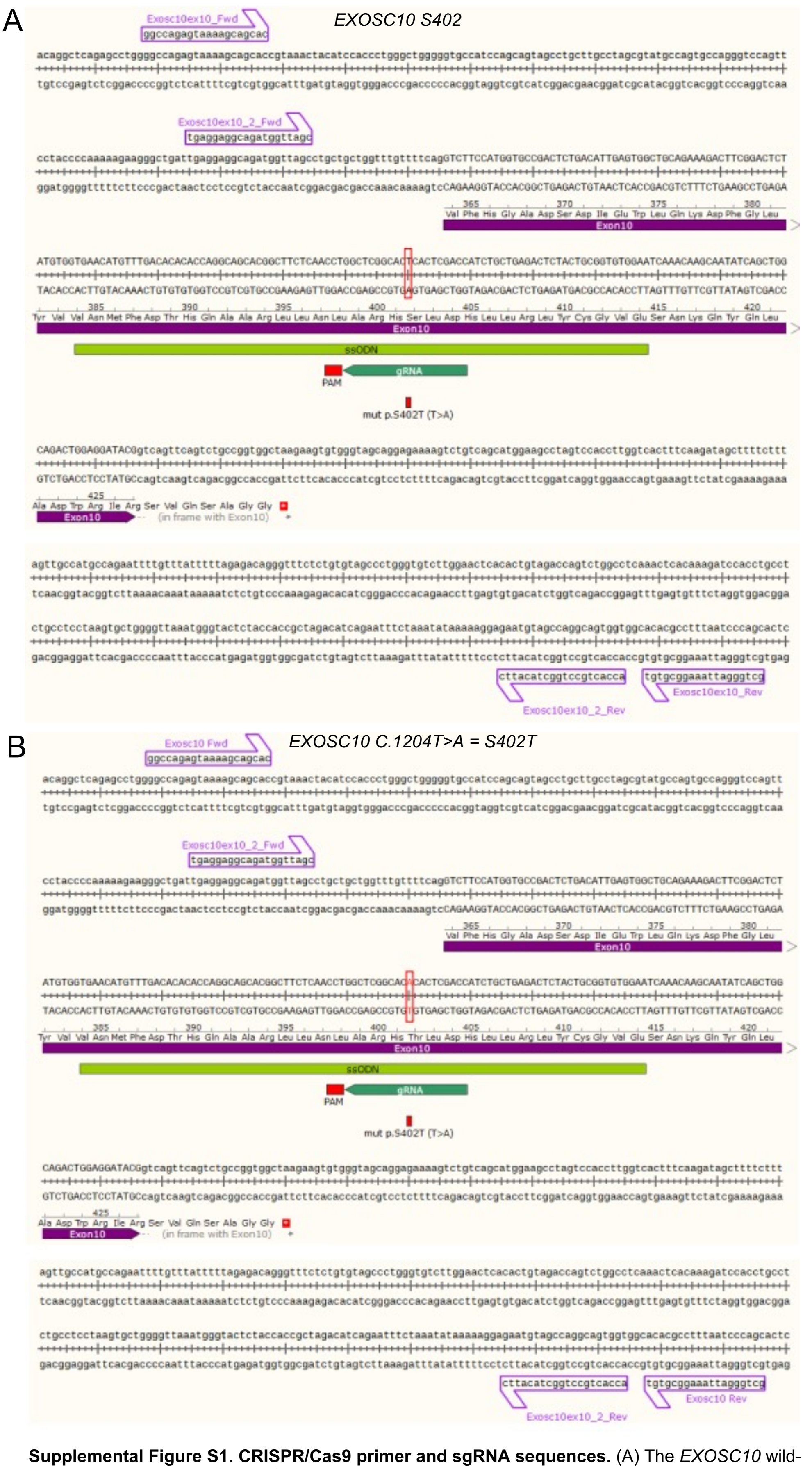

Supplemental Figure S1. CRISPR/Cas9 primer and sgRNA sequences. (A) The *EXOSC10* wild-type sequence relevant for the S402T mutation is shown. The base to be mutated is marked by a red rectangle. (B) The resulting mutant 1204T>A sequence is given.
