# Supplementary material for: Heterozygous loss‐of‐function alleles associate the conserved 3′‐5′ exoribonuclease EXOSC10 with hypersensitivity to the anticancer drug 5‐fluorouracil
Source: Mol Oncol. 2026 May 15:10.1002/1878-0261.70239. Online ahead of print. doi: 10.1002/1878-0261.70239 (PMC13398982; doi:10.1002/1878-0261.70239)
Supplement: Supplementary file 4 — Fig. S4. cBioPortal data for EXOSC10. [file MOL2-9999-0-s010.pdf]

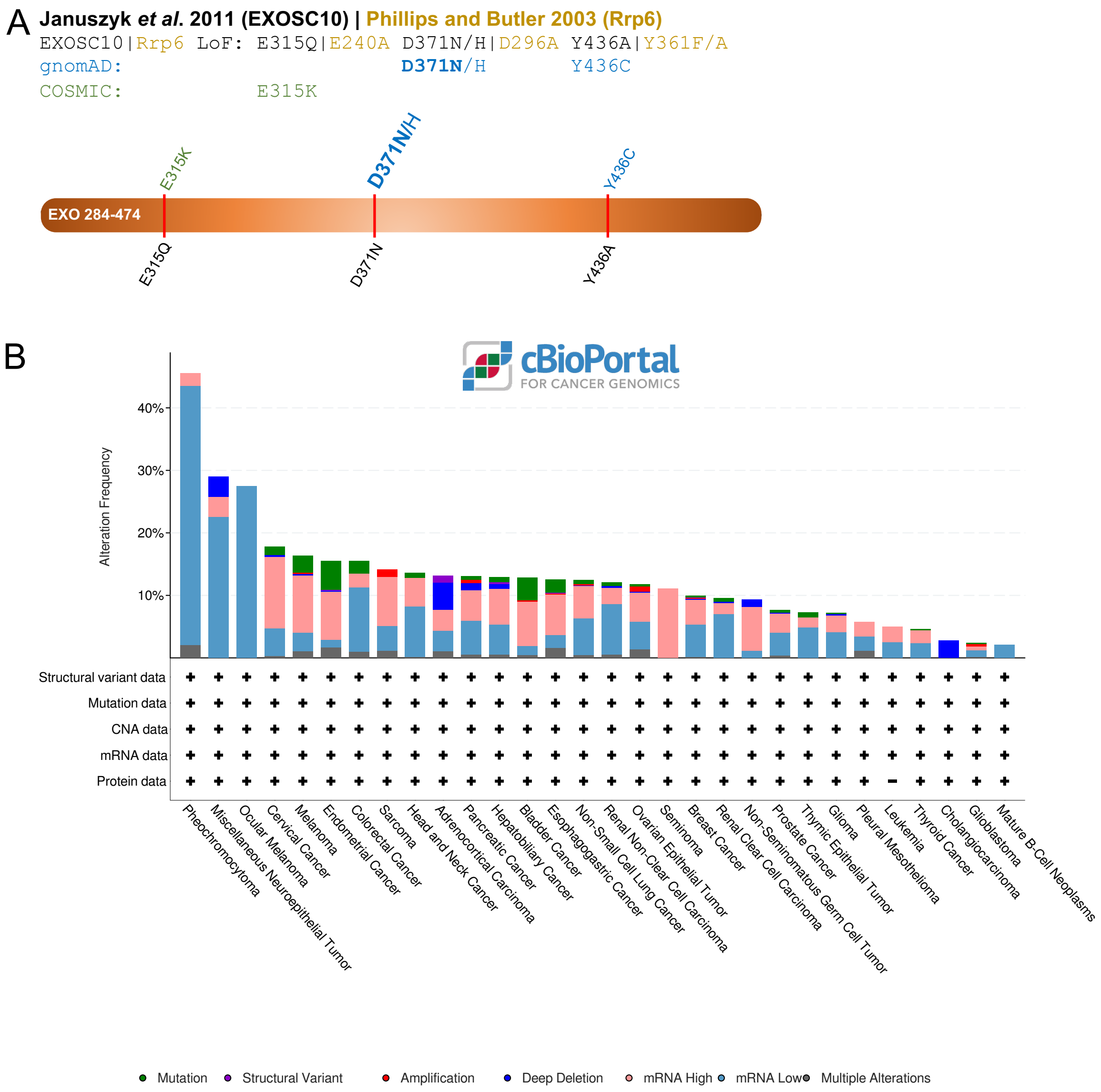

Supplemental Figure S4. cBioPortal data for EXOSC10. (A) References for conserved missense mutations in human EXOSC10 (black) and yeast Rrp6 (yellow) proteins are shown. Corresponding mutations referenced in gnomAD (blue) and COSMIC (green) are given. A schematic summarizes the references and positions of *EXOSC10* LoF alleles within EXOSC10's catalytic domain. (B) A color-coded bar diagram from cBioPortal plots the frequency and the data type (y-axis) against cancer samples (x-axis). A legend at the bottom summarizes the color code for alterations.
