# Supplementary material for: Heterozygous loss‐of‐function alleles associate the conserved 3′‐5′ exoribonuclease EXOSC10 with hypersensitivity to the anticancer drug 5‐fluorouracil
Source: Mol Oncol. 2026 May 15:10.1002/1878-0261.70239. Online ahead of print. doi: 10.1002/1878-0261.70239 (PMC13398982; doi:10.1002/1878-0261.70239)
Supplement: Supplementary file 5 — Fig. S5. gnomAD data for EXOSC10. [file MOL2-9999-0-s002.pdf]

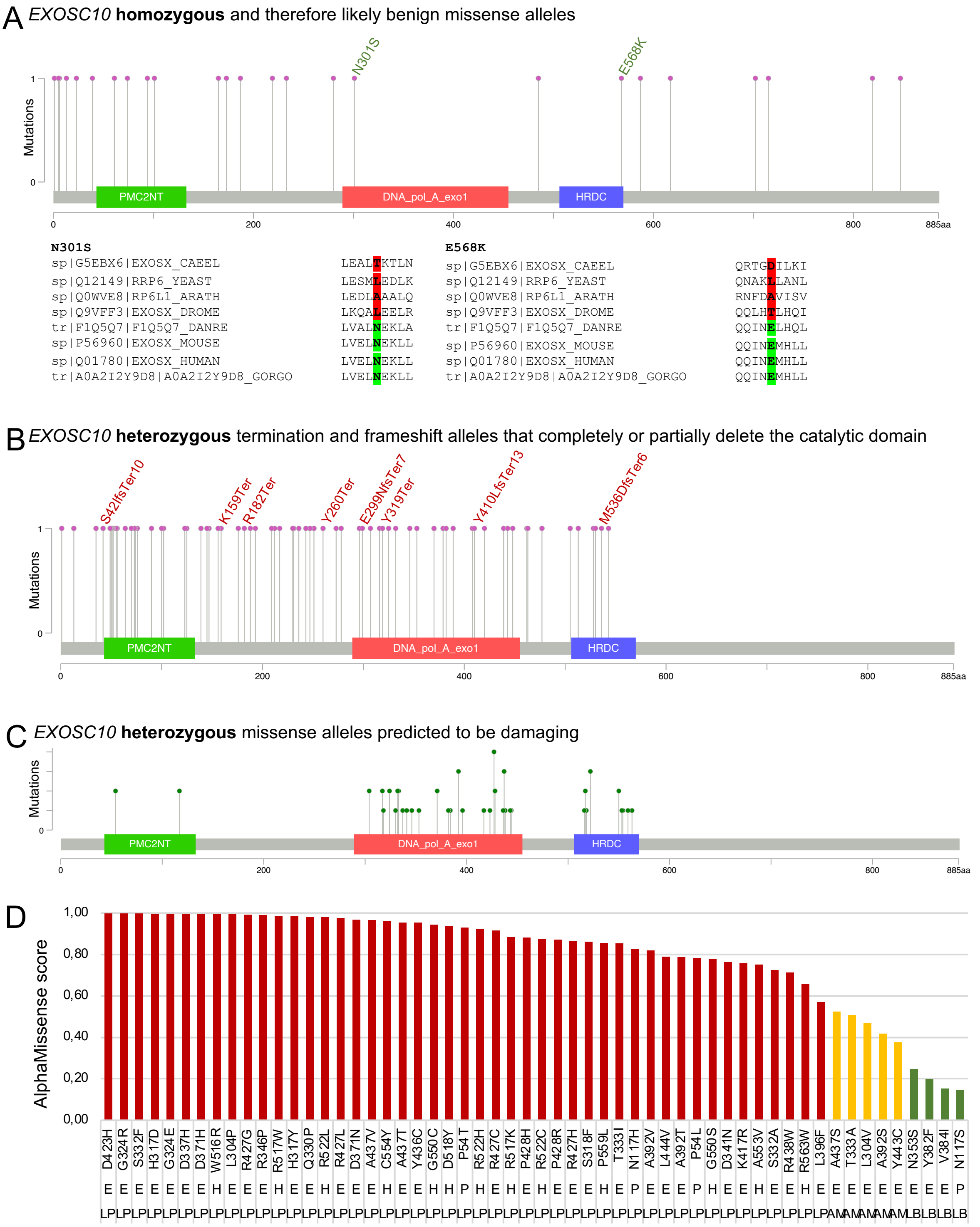

Supplemental Figure S5. gnomAD data for *EXOSC10*. (A) A lollipop plot at the top generated with cBioPortal's mutant maker tool shows the location of homozygous mutations (y-axis) and the protein sequence and domains (x-axis) within the primary sequence. Two mutations within the catalytic domains are indicated in green. A multiple sequence alignment generated with the EBI's MUSCLE algorithm is shown for selected amino acids from eight *EXOSC10* orthologs as indicated. Conserved and non-conserved residues are highlighted in green and red, respectively. (B) A lollipop plot shows the positions of selected frameshift (fs) and termination (ter) mutants in red within the amino acid sequence like in panel A. (C) A lollipop plot shows the number of alleles (y-axis) and the position of missense mutations predicted to be pathological (x-axis). (D) A color-coded bar diagram plots the AlphaMissense score (from 0 to 1; y-axis) against missense mutations (x-axis). Red, yellow and green indicate likely pathological (LP), ambiguous (AM) and likely benign (LB) mutations, respectively. The functional domains indicated at the bottom are PMC2NT (P), EXO (E) and HRDC (H).
